# Tumor-Derived Exosomes (TEX) and Their Role in Immuno-Oncology

**DOI:** 10.3390/ijms22126234

**Published:** 2021-06-09

**Authors:** Theresa L. Whiteside, Brenda Diergaarde, Chang-Sook Hong

**Affiliations:** 1 Department of Pathology and UPMC Hillman Cancer Center, University of Pittsburgh School of Medicine, Pittsburgh, PA 15213, USA; hongc@upmc.edu; 2 Department of Human Genetics and UPMC Hillman Cancer Center, University of Pittsburgh Graduate School of Public Health, Pittsburgh, PA 15213, USA; diergaardeb@upmc.edu

**Keywords:** cancer biomarkers, small extracellular vesicles (sEV), tumor-derived exosomes (TEX), TEX immune capture, immune suppression, TEX-mediated reprogramming

## Abstract

Extracellular vesicles (EVs) play a key role in health and disease, including cancer. Tumors produce a mix of EVs differing in size, cellular origin, biogenesis and molecular content. Small EVs (sEV) or **exosomes** are a subset of 30–150 nm (virus–size) vesicles originating from the multivesicular bodies (MVBs) and carrying a cargo that in its content and topography approximates that of a parent cell. Tumor-derived exosomes (TEX) present in all body fluids of cancer patients, are considered promising candidates for a liquid tumor biopsy. TEX also mediate immunoregulatory activities: they maintain a crosstalk between the tumor and various non-malignant cells, including immunocytes. Effects that EVs exert on immune cells may be immunosuppressive or immunostimulatory. Here, we review the available data for TEX interactions with immunocytes, focusing on strategies that allow isolation from plasma and separation of TEX from sEV produced by non-malignant cells. Immune effects mediated by either of the subsets can now be distinguished and measured. The approach has allowed for the comparison of molecular and functional profiles of the two sEV fractions in plasma of cancer patients. While TEX carried an excess of immunosuppressive proteins and inhibited immune cell functions in vitro and in vivo, the sEV derived from non-malignant cells, including CD3(+)T cells, were variably enriched in immunostimulatory proteins and could promote functions of immunocytes. Thus, sEV in plasma of cancer patients are heterogenous, representing a complex molecular network which is not evident in healthy donors’ plasma. Importantly, TEX appear to be able to reprogram functions of non-malignant CD3(+)T cells inducing them to produce CD3(+)sEV enriched in immunosuppressive proteins. Ratios of stimulatory/inhibitory proteins carried by TEX and by CD3(+)sEV derived from reprogrammed non-malignant cells vary broadly in patients and appear to negatively correlate with disease progression. Simultaneous capture from plasma and functional/molecular profiling of TEX and the CD3(+)sEV fractions allows for defining their role as cancer biomarkers and as monitors of cancer patients’ immune competence, respectively.

## 1. Introduction

Small extracellular vesicles (sEV), otherwise known as exosomes, have recently emerged as potential biomarkers in various human diseases [1,2]. In cancer, tumor-derived exosomes (TEX) are attracting attention as potential noninvasive biomarkers of tumor progression and of patient responses to immune therapies (IT) [3,4,5]. Specifically, it has been suggested that defective anti-tumor immune responses frequently seen in patients with advanced cancers are driven by TEX and may be responsible for tumor immune escape, unresponsiveness to IT, and resistance of tumors to oncological therapies [6,7,8]. 

Exosomes are a subset of extracellular vesicles (EVs) that differ from other EVs by their size, origin and molecular content. While the nomenclature for EVs has not been formally established, there is an agreement that small EVs (sEV) are distinct from larger EVs [9,10]. Exosomes are sEVs which according to current nomenclature include exomeres (<35 nm), small exosomes (Exo-S, 50–100 nm) and large exosomes (Exo-L, 100–150 nm) [7]. As exomeres fit into the particle rather than vesicle category, a suggestion has been made to replace the term “exosomes” and collectively name this sEV subset as “Extracellular Vesicles and Particles” or “EVPs” [11,12]. While differences in size and perhaps the molecular content are responsible for the heterogeneity of sEV and for the somewhat confusing nomenclature, one common feature that differentiates sEV from larger microvesicles (MVs: 500–1000 nm) and even larger apoptotic bodies (>1000 nm) is their cellular origin. Exosomes or sEV originate from the endocytic compartment of parent cells, are formed in late endosomes by the intraluminal invagination process and are assembled in the multivesicular bodies (MVBs). Their biogenesis has been extensively reviewed [13,14]. Exosomes are released into intercellular space after MVBs fuse with the plasma membrane of an exosome-producing cell. The endocytic origin of sEV endows them with distinct markers, such as TSG101, ALIX, flotillin, moesin, syntenin-1, CD9, and the surface topography that resembles the molecular content of their parent cells [15]. These attributes of exosomes, specifically their molecular and genetic signatures that are identical or similar to those of their parent cells, are a rationale for the current emergence of TEX as key components of the liquid tumor biopsy [4,16]. In addition, it appears that sEV produced by non-malignant cells, such as, e.g., immune cells in the tumor-microenvironment (TME), might undergo TEX-driven reprogramming and might also contribute to the tumor promotion [17] Activated immunocytes in the TME avidly produce sEV, which constitute a considerable proportion of total sEV in plasma of cancer patients [18,19]. While much attention has been devoted to TEX as potential cancer biomarkers and their contribution to cancer progression, immune cell-derived sEV are now emerging as equally important contributors to the unique tumor-orchestrated immune landscape in cancer. In this review, we first consider the evidence for the role of sEV play in tumor-induced immune suppression. Then, taking advantage of recent strategies for separation of TEX from non-TEX in plasma of cancer patients, we consider the role of sEV produced by immune cells in the tumor-bearing hosts as important participants in the downregulation of anti-tumor immune responses. The view that we champion is that in addition to TEX, immune cell-derived sEV induced by TEX are major participants in cancer-driven immune suppression and thus, in promoting cancer progression.

## 2. All sEV in Supernatants of Cultured Tumor Cells Are TEX

Much of what is currently known about TEX is based on analyses of EVs present in supernatants of cultured tumor cell lines [20,21,22,23]. As these cultures contained only tumor cells, all sEV isolated from their supernatants were TEX. This represented an obvious opportunity to learn about the molecular cargos and functional properties of TEX, including their interactions with immune cells. In experiments going back more than 15 years, we reported that TEX negatively regulated functions of the T-cell receptor (TcR) by inducing down-regulation in expression levels of the CD3-associated zeta chain. TEX were shown to be responsible for low TcR/zeta expression levels frequently seen in circulating T cells of cancer patients, especially those with advanced malignancies [24,25,26]. For example, decreased TcR zeta chain expression on circulating and tumor-infiltrating T cells have been linked to immune dysfunction and poor outcome in patients with breast cancer [27]. Down-regulation of the TcR zeta chain expression levels was consistently observed when human primary T cells from the peripheral circulation of healthy donors were co-incubated with TEX [28]. The IL-2R, another key receptor in T cells, was also negatively modulated by co-incubation with TEX, as reported by Clayton et al. [29]. We reported that expression and phosphorylation of JAK in activated T cells was reduced following exposure to TEX [30]. Since the JAK pathway integrity is essential for functions of IL-2, IL-7 and IL-15, the cytokines sharing the γ chain of the IL-2R, down-regulation of JAK activity by TEX was detrimental to T-cell proliferation [26,30]. Indeed, suppression of T cell proliferation was the most frequently reported effect of TEX coincubation with activated T cells [31]. We also observed that while TEX suppressed proliferation of activated CD3+CD8+ T cells, they promoted proliferation of CD3+CD4+ T cells [31]. Subsequent studies by us and others showed that coincubation with TEX induced expansion and differentiation of primary CD4+ T cells into functional CD4+FOXP3+CD39+ regulatory T cells (Treg) [26,32]. Consistent with these data, TEX were found to increase STAT5 phosphorylation in activated CD4+ T cells and to inhibit STAT5 phosphorylation in activated CD8+ T cells, suggesting that uptake of TEX differentially modulated expression levels JAK and STAT in T lymphocytes [33]. Importantly, sEV produced by cultured non-malignant cells (e.g., fibroblasts) were not inhibitory and promoted proliferation of all T cells [26,33]. Perhaps the most dramatic effect of TEX on T cells is observed when activated CD8+T cells expressing CD95 or PD-1 on the cell surface are co-incubated with TEX carrying FasL or PD-L1 [26]. Within minutes, apoptosis of CD8+ T cells is complete, and early changes in the cell membrane (i.e., Annexin binding) are associated with caspase 3 cleavage, cytochrome C release from mitochondria, loss of mitochondrial membrane potential (MMP) and DNA fragmentation, suggesting the engagement of the extrinsic as well as intrinsic apoptotic cascades [30]. Having reported TEX-induced apoptosis of effector T cells, we went on to show that the PI3K/AKT pathway is the key target for TEX in activated CD8+ T cells. We demonstrated that dramatic, time-dependent AKT dephosphorylation and concomitant decreases in expression levels of BCL-2, BCL-xL and MCL-1 accompanied by an increase in levels of pro-apoptotic BAX occurred in these T cells during co-incubation with TEX [30]. 

T lymphocytes are not the only immune cells targeted by TEX. Other immune cells, including human NK cells, B cells and monocytes uptake and internalize TEX more effectively than T cells [34], and co-incubation with TEX also alters their activities [35]. TEX which carry MICA and MICB ligands down-regulated expression of the activating receptors, especially NKG2D, in NK cells [36]. Activation and cytotoxicity of NK cells are in part mediated by transforming growth factor β (TGF-β), which is prominently displayed on TEX as transforming growth factor-latency associated protein (TGF-LAP), the form necessary for TGF-β activation upon binding to integrins, e.g., α6βV, on the surface of recipient cells [36,37]. TEX, which are known to carry CD39 and CD73 ectonucleotidases are able to make adenosine from ATP [38] and can induce suppression of activated B cells, because adenosine can convert activated B cells into regulatory B cells [39]. TEX have been reported to inhibit normal differentiation of monocytes and to convert monocytes into TGF-β-expressing DCs, which secreted prostaglandin E_2_ (PGE_2_) and interfered with the generation of cytolytic T cells [40,41]. Further, TEX are implicated in skewing the differentiation of myeloid precursor cells directing it toward the development into highly suppressive MDSCs [41,42]. This function of TEX was dependent on MyD88 signaling in monocytes and the presence of TGF-β and PGE_2_ in the TEX cargo [42]. Interestingly, TEX-induced Treg proliferation and increased suppressor functions of proliferating T reg were associated with increased production of adenosine or inhibitory cytokines (TGF-β, IL-10) by Treg co-incubated with TEX [32]. Thus, TEX carry the machinery to induce production of inhibitory cytokines, adenosine or PGE_2_ [32,43], and thereby indirectly influence functions of cells neighboring immunocytes interacting with TEX.

These initial in vitro and ex vivo studies of TEX were later supplemented by in vivo experiments, whereby human TEX were injected into healthy or tumor-bearing mice [44,45,46,47]. Remarkably, human sEV injected IV or IP into mice were not rejected, and in tumor-bearing mice, they promoted tumor development or progression that was associated with significantly reduced infiltration of immune cells into the tumor mass [47]. In healthy, non-tumor bearing mice, TEX had no detectable adverse effects on functions of immune cells [44]. 

Studies reported in the literature performed with TEX produced by cultured tumor cells have established that TEX are biologically active in vitro and in vivo and are capable of modulating functions of different types of immune cells by various direct or indirect mechanisms. However, while TEX produced by cultured tumor cells share the common origin, they are highly heterogeneous representing small and large exosomes derived from MVBs, microvesicles (MVs) formed by “budding” of the tumor cell surface membrane, and apoptotic bodies of various sizes with variable contents. Most of the reported studies did not discriminate between the various categories of sEV subsets present in culture supernatants; therefore, it remains unclear whether all TEX share the above described attributes or whether a “division of labor” among TEX exists and only some, but not all, TEX induce immune suppression that contributes to cancer immune escape.

## 3. Cancer Patients’ Plasma Is a Complex Mix of Various sEV Types 

In the tumor-bearing hosts, body fluids, including plasma, contain very large numbers of vesicles which originate from various circulating as well as tissue-resident malignant and non-malignant cells [20,48,49,50]. This mix of various vesicles containing exomeres, small and large exosome subsets, MVs, oncosomes and apoptotic bodies [10] is clearly more heterogenous than are sEV found in supernatant of a cultured tumor cell line. Therefore, sEV, representing a subset of vesicles with the 30–150 nm diameter and the unique endosomal biogenesis, have to be isolated and separated from other vesicles if their numbers in the circulation, molecular content and functions are to be discerned.

A variety of methods for sEV isolation have been described and are currently available. [49,51,52,53]. Density gradient ultracentrifugation at 100,000× *g* for several hours remains the most widely used method despite its various limitations as reviewed [52,54]. We have adapted size exclusion chromatography (SEC) preceded by differential centrifugation and ultrafiltration for sEV isolation in a relatively short Sepharose column that processes 1mL volumes of plasma in minutes and elutes the bulk of sEV in PBS in fraction #4 as previously reported [55]. The eluted sEV are morphologically intact, non-aggregated, partly but not entirely depleted of “contaminating” plasma proteins and functionally competent, as determined in co-incubation assays with primary human immune cells [56,57].These attributes of isolated sEV are critically important for their subsequent fractionation by immune capture [58]. We recover from 1 × 10^10^ to 1 × 10^12^ sEV per 1 mL of pre-cleared cancer plasma processed by SEC [17]. Recovered sEV are tested for the protein content and for vesicle numbers (NanoSight), vesicular morphology by transmission electron microscopy (TEM), molecular content as well as the presence of markers confirming their endocytic origin (Western Blots). Characteristics of the sEV isolated by our differential centrifugation/ultrafiltration/SEC method meet the criteria established by the ISEV guidelines for the sEV characterization [9]. 

**Figure 1:**
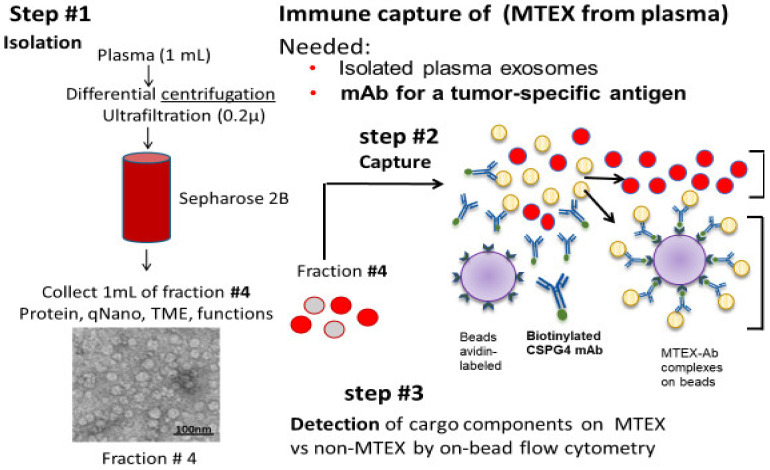
Immune capture with biotinylated anti-CSPG4 Abs of melanoma cell-derived exosomes on streptavidin-labeled beads from plasma of patients with melanoma. Exosomes were first isolated from plasma by size exclusion chromatography (SEC; step #1). Fraction #4 containing a mix of melanoma cell-derived vesicles (MTEX) and vesicles derived from non-malignant cells (NMTEX) were immuno-captured (step #2) Non-captured exosomes (NMTEX) in red were re-captured on beads with anti-CD63 mAbs for detection by on-bead flow cytometry (step #3). Two different clones of CSPG4 mAb (763.74 and 225.28) specific for distinct epitopes of this protein were used for capture and detection, respectively. Reproduced with changes from Sharma et al., Ref # 58.

Total sEV isolated from plasma of cancer patients by SEC or other methods are expected to be enriched in TEX. Compared to non-malignant cells, tumor cells produce much larger numbers of sEV, which accumulate in body fluids, and as indicted above, these sEV represent a mix of TEX and sEV produced by non-malignant cells (non-TEX). Therefore, a strategy is needed to separate and differentiate TEX from non-TEX and to profile the molecular content or study functions of these two sEV fractions. We selected to use immune-based capture, whereby mAbs specific for proteins carried by TEX but not by non-TEX are used for vesicle separation [58]. The TEX/non-TEX isolation strategy combining 3 different steps is illustrated in Figure 1 [58]. Using plasma of patients with melanoma as a source of sEV isolated by SEC in step1, this strategy makes use of mAbs specific for an epitope of Chondroitin Sulfate Proteoglycan-4 (CSPG4), which is overexpressed on most melanoma cells and on melanoma cell-derived sEV but not on non-malignant cells or sEV these non-malignant cells produce [59,60,61]. Few such Abs with specificity for a tumor associated antigen (TAA) are available except, of course, for Abs with specificity for mutated tumor proteins. Thus, mAbs specific for CSPG4, otherwise known as “High Molecular Weight Melanoma-Associated Antigen, generated and tested by Dr. Soldano Ferrone (currently at Harvard University), represents a unique melanoma-specific, rigorously characterized reagent [62]. It can be used for immune capture of melanoma cell-derived exosomes (MTEX) and is available on request from Dr. Ferrone. Using biotinylated anti-CSPG4 Abs, we captured MTEX on streptavidin beads, separating them from non-MTEX (non-malignant cell-derived sEV) which remained in solution (Figure 1). After non-MTEX were recaptured on beads using anti-CD63 mAbs, the two separated sEV fractions were then evaluated in step 3 by quantitative on-bead flow cytometry for molecular components carried on their surface and for their functions by coincubation with primary immune cells obtained from peripheral blood of healthy donors (HDs) as previously described [58]. The multi-step process of sEV isolation, capture and antigen detection in cancer plasma requires substantial technical expertise, Abs specific for TAAs and extensive reproducibility measures to make it applicable to monitoring of clinical specimens. Attempts to simplify this immunocapture process by additions of capture Abs directly to the plasma were not successful in our hands. We suspect that sEV isolation by ultrafiltration and SEC yielding sEV that are mostly, albeit not entirely, free of “contaminating” plasma proteins facilitates interactions of capture Abs with sEV. 

## 4. TEX and Non-TEX Isolated from Cancer Patients’ Plasma Have Distinct Profiles 

As indicated above, studies of TEX produced by cultured tumor cell lines consistently indicated their enrichment in a broad variety of immunosuppressive proteins [63]. In contrast to TEX in supernatants of cultured tumor cells, sEV isolated from cancer plasma contained tumor cell derived TEX and sEV derived from various non-malignant cells. For the first time, it became possible to inquire which of the two sEV fractions was responsible for immune suppression in cancer. The phenotypic and functional evaluations of sEV recovered from melanoma patients’ plasma and separated into MTEX and non-MTEX represent an attempt to gain insights into and compare the potential of each fraction to induce changes in immune cells. Before this could be done, extensive evaluation of the immunocapture process was performed to document its efficiency for a complete and reproducible separation of the two sEV fractions [64]. Indeed, MTEX were found to carry a profile of several melanoma associated antigens (MAAs), including CSPG4, while non-MTEX did not carry MAAs [58,64]. The proteins present on sEV surface of the separated subsets were quantified by on-bead flow cytometry and expressed as relative fluorescence intensity (RFI) values [65]. Stimulatory/inhibitory functions were measured in coincubation assays of primary activated human T cells or NK cells +/− sEV, and the stimulatory/suppressive (stim/supp) ratios of the surface proteins were determined [64].

In view of the data reported above, analysis of the MTEX molecular content was not a surprise. The enrichment in immunosuppressive proteins, such as PD-L1, TGF-β, CTLA-4, CD39/CD73 or TRAIL, as well as paucity of immunostimulatory proteins, such OX40L or OX40, were in agreement with data previously reported by us and others for TEX from tumor cell supernatants [57,66]. MTEX had significant immunosuppressive activity (i.e., they down-regulated CD69 expression on CD8+ T cells, inhibited effector cell proliferation, induced effector cell apoptosis). The MTEX suppressor score (calculated as a mean RFI value) correlated with total sEV protein levels in plasma (*p* = 0.002). Furthermore, the ratios of MTEX/total sEV levels in plasma varied broadly among patients with melanoma, and significantly correlated with the MTEX capability to induce apoptosis in activated CD8+ T cells [64]. This comparative phenotypic and functional analysis of MTEX and non-MTEX with total plasma sEV revealed that while mean values for all endpoints in total plasma sEV and MTEX correlated, non-MTEX values were significantly different. At the same time, we noted that non-MTEX were phenotypically and functionally distinct from sEV in plasma of HDs tested in parallel assays. We had expected that non-MTEX derived from non-malignant cells in patients with melanoma would resemble sEV in plasma of HDs. Instead, in every functional assay, non-MTEX did not match activity levels of sEV in HDs, although phenotypically and functionally they significantly differed from paired MTEX. This comparative study of paired exosome samples indicated that sEV produced by non-malignant cells in cancer patients were phenotypically and functionally distinct from sEV produced by non-malignant cells of HDs. Even more striking was the realization that non-MTEX could induce apoptosis of CD8+ T cells, and that levels of this apoptosis positively correlated with disease stage at diagnosis [64]. Further, significant negative correlation of disease stage and the stimulatory/suppressive (stim/supp) protein ratio of non-MTEX was observed at *p* < 0.0007, suggesting that immune regulation by non-MTEX had an impact on disease stage at diagnosis. While preliminary, these data uncovered the potential of both MTEX and non-MTEX to alter functions of immune cells depending on the ratios of stim/supp proteins present in their cargo. Although MTEX in melanoma patients’ plasma are largely responsible for immune suppression, as previously observed with sEV derived from cultured tumor cell lines, non-MTEX are not silent. The mean stim/supp ratio was 0.6 for MTEX, 1.4 for non-MTEX and 2.2 for HDs’ sEV, a clear indication that non-MTEX are functionally distinct from sEV in plasma of HDs and from MTEX [64].

## 5. Mechanisms Involved in sEV-Mediated Immune Suppression in Cancer

We and others have reported earlier that sEV isolated from supernatants of tumor cell lines or cancer plasma and co-incubated with activated T cells delivered negative signals that reduced expression levels of key proteins involved in activation (e.g., CD69) or proliferation (e.g., the T cell receptor (TCR) associated zeta chain; JAK/STAT signaling) of immunocytes [24,25,26,27,28]. Mechanisms through which TEX alter functions of recipient immune cells are incompletely understood. Using one or more mechanisms enabling the cell entry, such as membrane fusion, phagocytosis, endocytosis, integrin-mediated adhesion or receptor/ligand signaling, TEX deliver their cargo to recipient cells [67]. Various immune cells differ in their ability to internalize and process TEX. T cells appear to mainly interact with TEX via the receptor/ligand-mediated signaling, and unlike other mononuclear cells do not readily internalize TEX [34]. Instead, TEX interacting with surface proteins expressed on T cells deliver signals which initiate a Ca^2+^ flux and activate downstream signaling, resulting in alterations of the recipient cell transcriptome and re-programing of T-cell functions [34]. Other immunocytes avidly uptake and internalize TEX [34]. Thus, NK cells were highly susceptible to TEX, which in a dose-dependent manner inhibited surface expression of NKG2D and blocked NK cell lytic activity against tumor cell targets [57]. Coincubation of dendritic cells (DC) with TEX was characterized by their rapid internalization that resulted in the downregulation of CD80 and CD86 on the DC surface and inhibited DC maturation [31]. It is likely that following internalization, TEX deposit their membrane-protected content, including mRNA, miRNA and DNA, inside recipient cells. Upon further processing, these molecules might integrate into the cell machinery to initiate recipient cell re-programming, which alters the recipient cells transcriptome and proteome as previously reported [35]. A best example of the TEX ability to alter functions of recipient cells is re-programming of the bone marrow microenvironment by melanoma cell-derived TEX reported by Peinado et al. [68]. These TEX upon in vivo transfer to the murine bone marrow transformed it into a pro-metastatic niche promoting the development of melanoma and interfering with normal hematopoiesis. Multiple recent studies confirm the ability of TEX to directly alter functions of various recipient cells, including immune cells [69,70]. 

**Figure 2:**
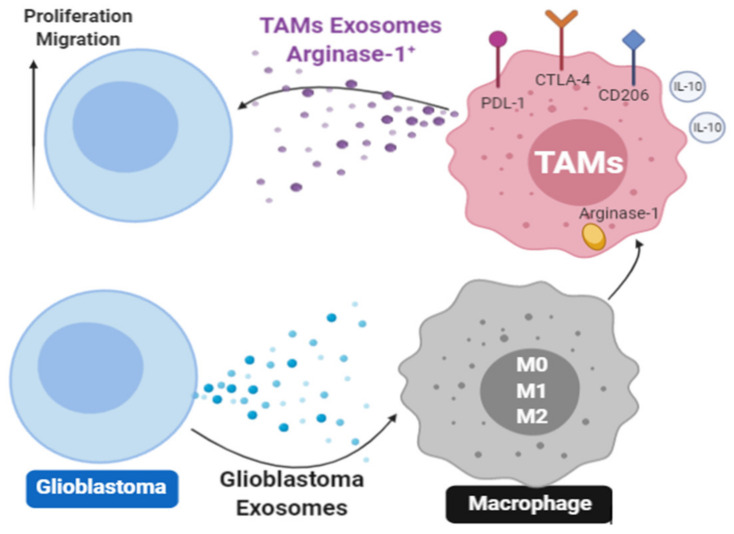
Exosomes produced by glioblastoma cells are taken up by non-malignant macrophages present in the TME and reprogram them by driving their differentiation into tumor associated macrophages (TAMs). Expression levels of inhibitory ligands on the TAM surface, production of arginase 1 and IL-10 by TAMs and secretion of TAM-derived exosomes decorated with arginase 1 and carrying IL-10 are upregulated. TAM-derived arginase-1+ exosomes inhibit anti-tumor immune cells in the TME and drive glioblastoma proliferation. The figure was prepared by Dr. Azambuja based on the published data in reference [69].

Indirectly-mediated immune suppression orchestrated by TEX has also been reported [33]. It appears to be implemented either by TEX-driven skewing/arrest of immune cell differentiation [71] or by TEX-induced de novo production and release by activated T cells of immunosuppressive sEV, the process we refer to as “TEX-driven reprogramming” [17]. Among various mechanisms TEX use for skewing or arrest of immunocyte differentiation is the inhibition of the antigen processing machinery (APM) components, such as TAP1, upon coincubation of matured DC with TEX [31]. An example of TEX-driven reprogramming was recently provided by Azambuja et al., using TEX produced by cultured glioblastoma cells [69]. Figure 2 shows that glioma cell-derived TEX can convert macrophages (M0, M1 or M2) to highly suppressive tumor-associated macrophages (TAMs) which up-regulate surface expression of immunosuppressive ligands as well as release of exosomes enriched in arginase 1and IL-10. These “secondary” TAM-derived exosomes not only have the means to suppress immune cells but also actively promote glioblastoma growth by supplying arginase1 to the tumor [69].

**Figure 3:**
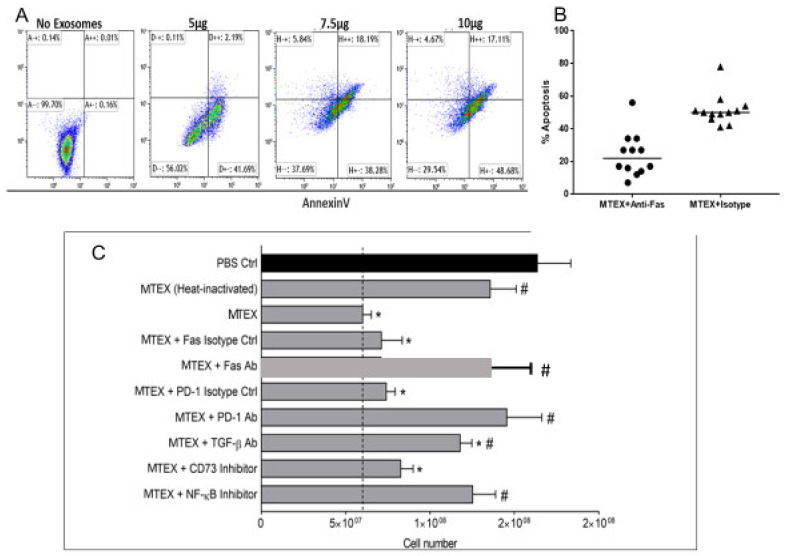
MTEX induced suppression of T cell proliferation or apoptosis measured in coincubation assays was blocked by various Abs or pharmacologic agents able to disrupt the specific receptor/ligand binding. In (**A**), dose-dependent apoptosis of human primary CD8+ T cells by MTEX after 6h coincubation. In (**B**), inhibition of MTEX-induced apoptosis in the presence of anti-Fas Abs relative to isotype control. Note the partial inhibition of apoptosis by anti-Fas Abs. In (**C**), activated human CD8+ T cells were pre-treated with blocking Abs or pharmacological inhibitors for 1 h followed by 72 h coincubation with MTEX. T cell numbers were counted using a flow cytometer. The dotted line indicates MTEX-induced suppression of T cell proliferation. Note simultaneous blocking of T cell proliferation by antagonists of different signaling pathways. Data are means ± SD of triplicate cocultures. * *p* < 0.05 vs. PBS control and # *p* < 0.05 vs. MTEX alone. Reproduced and modified with permission from Supplemental Files in reference [64].

Importantly, our sEV separation experiments showed that almost all of the above described direct and indirect effects TEX induce in immune cells could be reproduced with MTEX isolated from plasma of melanoma patients and also, to a variable extent, with non-MTEX [58,64]. Figure 3A shows that MTEX-mediated apoptosis of activated CD8+ T cells was concentration dependent, occurred in >60% of T cells and was partially but significantly blocked by anti-Fas Abs (Figure 3B). Further, in Figure 3C, blocking of MTEX-induced CD8+ T cell proliferation by various Abs and pharmacological inhibitors is shown to illustrate the potential involvement of various signaling pathways (Fas/FasL; TGF-βR/TGF-β; PD-1/PD-L1; adenosine (CD39/CD73) and NF-κB) in MTEX signaling. We have also shown that MTEX-induced downregulation of CD69 in activated CD8+ T cells rapidly translated into changes in mRNA transcripts for CD69, suggesting that MTEX-driven surface signaling leads to transcriptional activation [64]. We showed that suppression of T-cell or NK-cell functions by MTEX was concentration dependent, was absent when sEV were pre-treated with proteinase K or denatured and was reversed by: (a) neutralizing Abs to inhibitory ligands (FasL, TRAIL, PD-L1, CTLA-4, MICA/B); (b) blocking of MTEX uptake by recipient cells; and, (c) pharmacologic inhibitors of TGF- β, adenosine, AKT/PI-3K or NF-κB pathways [64]. The amelioration in vitro of sEV-mediated immune cell suppression simultaneously but not completely by several different inhibitors suggests that not one but many molecular pathways may be engaged when TEX are interacting with T cells [56].

**Figure 4:**
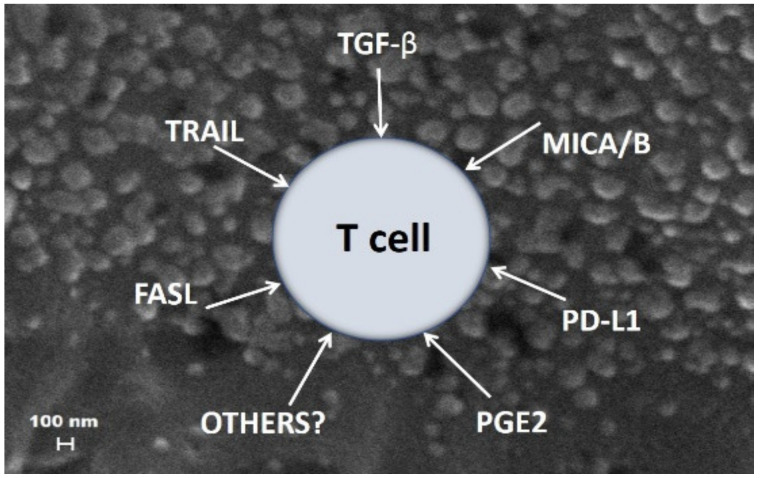
An imaginary view of multiple sEV in plasma of a cancer patient. Tumor-derived sEV such as MTEX carry various immunosuppressive cargos and interfere with anti-tumor functions of activated T cells in the circulation. Note that the T cell found in the midst of sEV is simultaneously subjected to different inhibitory signals that engage multiple suppressive pathways, leading to loss of T cell functions and/or to T cell apoptosis. Reproduced from ref [56].

Based on these data, MTEX emerge as major contributors to tumor-associated immune suppression and thus to tumor progression. Billions of MTEX whose surface is decorated with multiple inhibitory proteins circulate freely in cancer patients’ plasma. They can simultaneously deliver multiple suppressive signals to immune cells (see Figure 4), inducing loss of functions and apoptosis of activated T or NK cells, which are highly susceptible to MTEX-mediated apoptosis [64]. MTEX deliver inhibitory signals to the surface of effector immune cells responsible for anti-tumor activity and may kill activated immune cells. However, MTEX also reprogram other immune cells by initiating signaling cascades that activate various endogenous molecular pathways [34] and/or re-organize transcription in immune cells by utilizing miRNAs [70]. Further, MTEX mediate autocrine signaling, supporting tumor progression and metastasis and are involved in the epithelial-mesenchymal transition [72]. There is preliminary evidence that non-MTEX also participate in direct and indirect reprogramming of immune cells, and their role in immune regulation is under investigation, as described below.

## 6. Plasma sEV Derived from Non-Malignant Cells Are Reprogrammed to Mediate Immune Suppression in Cancer 

A likely possibility that non-malignant sEV contribute to tumor-induced immune suppression can be directly tested by using the immunocapture strategy with biotinylated anti-CD3 mAbs for sEV capture, as previously described [19]. The strategy is analogous to that illustrated in Figure 1 for sEV capture using anti-CSPG4 mAbs, and it demonstrates that Ab-based immune capture can be successful with other Abs and plasma sEV derived from cells other than tumor cells. Since only T cells express CD3, sEV that are produced by T cells can be separated from CD3(−) sEV and recovered on beads. We were surprised to learn that CD3(+) sEV represented >50% of total plasma sEV in some patients with head and neck squamous cell carcinoma (HNSCC) [19]. Even more interesting was a finding that both CD3(+) and CD3(−) sEV fractions were enriched in immunosuppressive proteins such as PD-L1, CTLA-4, COX2 or CD39/CD73 ectonucleotidases. Further, patients with high levels (i.e., above the mean level for the entire cohort) of immunosuppressive proteins in CD3(+) sEV had stage III/IV tumors and positive lymph nodes, while patients with low levels of the suppressive cargo in CD3(+) sEV had less advanced (stage I/II, N0) disease [19]. In HNSCC patients with early stage I/II disease, CD3(+) sEV carried high levels of immunostimulatory cargo (i.e., had high expression of OX40 or OX40L), while in patients with advanced disease and positive lymph nodes, CD3(+) sEV had low levels of OX40 or OX40L, and in coincubation assays induced apoptosis of CD8+ effector T cells and promoted expansion of Treg [19]. Interestingly, CD3(−) sEV fractions were significantly enriched in tumor-associated proteins such as CD44v3 [73] and thus were presumably largely derived from tumor cells [74]. The high expression levels of inhibitory proteins and low expression levels of OX40 and OX40L in CD3(−) sEV fractions were also associated with advanced disease [73]. These data provided preliminary evidence that the molecular content of both CD3(−)(*TEX* enriched) and CD3(+)(*T-cell* derived) sEV fractions correlated with disease activity and disease progression in patients with HNSCC. Potentially, both CD3(−) sEV and CD3(+) sEV isolated from the same blood sample could be utilized as biomarkers of tumor progression in patients with HNSCC. Importantly, analogous conclusions were made by investigators who used anti-CD45 mAbs for capture of mononuclear cell-derived CD45(+) exosomes and their separation from tumor-enriched CD45(−) sEV [18]. 

## 7. Plasma sEV as Monitors of Cancer Patients’ Responses to Oncological Therapy 

Data accumulated in the literature and our published results provided preliminary evidence that immunosuppressive sEV impact cancer patients’ responses to immunotherapy (IT) [75,76,77]. We have reported that sEV isolated from plasma of cancer patients carry a rich cargo of immunosuppressive proteins, which inhibit functions of normal human immune cells in vitro and in vivo and correlate with disease activity and progression [43]. Specifically, we reported that PD-L1 levels on sEV from HNSCC patients’ plasma correlate with lymph node positivity, tumor stage and disease activity [77]. Only sEV with high levels of PD-L1 suppressed T-cell functions, and anti-PD-1 mAbs reversed inhibitory activity of PD-L1+ sEv [77]. Since this initial report, several other investigators confirmed the important role of PD-L1+ EVs in cancer and cancer IT [76,78,79].

**Figure 5:**
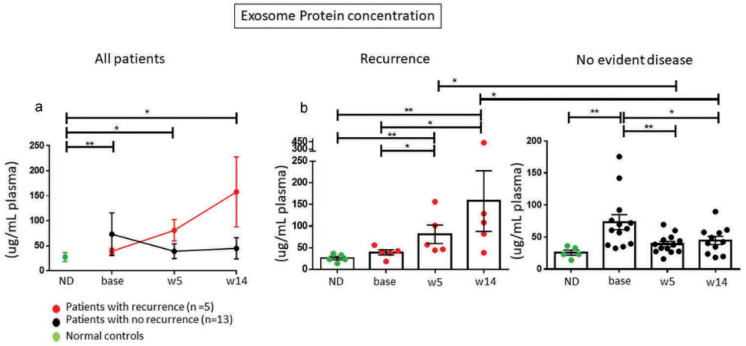
Changes in plasma exosomes (shown as total sEV protein level) of HNSCC patients treated with cetuximab/IMRT/ipilimumab. Five patients had recurrence and 13 were NED at 2 yrs. after therapy. Note decreased exosome protein in patients who remained NED for >2 yrs. * *p* = 0.05; ** *p* = 0.005. ND= normal donors. Reproduced from ref [80].

To assess the potential role of TEX and CD3(+) sEV in plasma of cancer patients as biomarkers able to predict patients’ response to oncological therapies, we serially monitored molecular profiles of these sEV subsets in a small cohort of HNSCC patients with recurrent/metastatic disease treated with cetuximab, IMRT and ipilimumab in a phase I clinical trial (NCT01935921) [80]. TEX and CD3(+) *non*TEX in plasma of HNSCC patients were isolated from plasma pre, during and post therapy and monitored. The patients with early recurrence (*n* = 5) had higher plasma total sEV levels, higher TEX/total sEV ratios and higher levels of CTLA4+, PD-L1+ and CD15s+ exosomes during and after immuno-radiotherapy than the 13 patients who remained disease free >2 years. after therapy (Figure 5) [80]. Using immunocapture with anti-CD3 mAbs, we showed that CD3(+) T-cell-derived sEV in patients with recurrence were also highly enriched in the same immunosuppressive proteins, while CD3(+) sEV in responders were not. The TEX/total sEV ratio in plasma proved to best discriminate patients who recurred from those who remained disease free [80]. These data suggested that changes in levels and cargos of circulating sEV reflect responses of cancer patients to oncologic therapies. In a highly quoted *Nature* paper, high plasma levels of PD-L1+ exosomes in patients with melanoma were reported to associate with poor responses to anti-PD1 therapy [75]. Another recent contribution by Cordonnier et al. reports that circulating exosomal PD-L1 levels measured in melanoma patients pre and post therapy correlated with response to therapy and predicted PFS and OS [81]. Thus, our data and those reported in the literature support the hypothesis that immunosuppressive plasma sEV interfere with cancer patients’ responses to immuno-oncologic therapies and promote tumor progression. Further, our published data indicate that in patients with advanced melanoma or HNSCC, TEX represent the major circulating exosome subset contributing to immune suppression and cancer progression [5,26,35,82]. At the same time, the data suggest that reprogrammed non-malignant sEV, including CD3(+) sEV, also contribute to cancer-driven immune suppression and, like CD3(−) TEX might be predictive of response to oncological therapies. 

## 8. Future Directions

The remarkable recent progress in immunotherapy of solid cancers with ICIs has called attention to a long-neglected role of the host immune system in regulating cancer progression and response of patients to oncologic therapies. It is well documented that advanced metastatic disease is associated with profound immune dysfunction, which is characterized by high numbers of circulating immunosuppressive sEV. In this review, we have summarized the evidence showing that these circulating sEV, including TEX and non-TEX, carry and deliver messages relevant to patients’ immune competence and thus to tumor activity, stage, grade and migration capability. Immunosuppressive sEV circulating in cancer patients’ body fluids regulate anti-tumor responses of immune effector cells and thus exert impact on disease progression and on patients’ responses to oncologic therapies. The quality and quantity of molecular messages sEV carry can now be measured in TEX and non-TEX fractions, serving as potential cancer as well as immune competence biomarkers. Extensive reprogramming by TEX of non-malignant cells in the TME represents a vicious cycle that leads to ever greater immune suppression mediated by TEX and non-TEX produced by reprogrammed immune cells. Our data suggest that TEX and sEV produced by non-malignant cells have a potential to qualify as non-invasive biomarkers of cancer progression and response to therapy. These data are preliminary and require validation in future experiments and in clinical trials. An opportunity for capturing TEX and CD3(+) sEV from the same plasma specimen as described above, extends the list of potentially informative messages plasma sEV carry in their cargo. Currently, the challenges of fitting or decoding the messages in sEV cargo and correlating them with clinical information remain the major aspect of the ongoing biomarker validation process. We are confident that technical challenges of working with sEV will be successfully solved in the near future and that TEX and non-TEX serving as a liquid biopsy will contribute to cancer diagnosis, prognosis and monitoring responses to oncological therapies.
